# Hydrogen bonds are a primary driving force for *de novo* protein folding. Corrigendum

**DOI:** 10.1107/S2059798318004047

**Published:** 2018-04-06

**Authors:** Schuyler Lee, Chao Wang, Haolin Liu, Jian Xiong, Renee Jiji, Xia Hong, Xiaoxue Yan, Zhangguo Chen, Michal Hammel, Yang Wang, Shaodong Dai, Jing Wang, Chengyu Jiang, Gongyi Zhang

**Affiliations:** a Department of Biomedical Research, National Jewish Health, Denver, CO 80206, USA; b Department of Immunology and Microbiology, School of Medicine, University of Colorado Denver, Aurora, CO 80206, USA; c Department of Chemistry, University of Missouri, Columbus, Mississippi, USA; d Physical Biosciences Division, Lawrence Berkeley National Laboratory, Berkeley, CA 94720, USA; e Department of Biochemistry and Molecular Biology, Peking Union Medical College, Beijing 100005, People’s Republic of China

**Keywords:** hydrogen bonds, *cis*/*trans*-proline, protein folding, corrigendum

## Abstract

The paper by Lee *et al.* [(2017). *Acta Cryst.* D**73**, 955–969] is withdrawn.

We wish to withdraw the paper by Lee *et al.* (2017 ▸) on the crystal structure of a protein believed to have been AID (activation-induced cytidine deaminase). It has subsequently been shown that the crystal studied was of *E. coli* RNA-binding protein Hfq, a contaminant in the protein preparation. The associated PDB entry, 5w09, has also been made obsolete. Our conclusions regarding the critical role of proline residues in protein folding, successful folding of proteins at high pH, and hydrogen bonds as a driving force in *de novo* protein folding are not affected, and further details will be published elsewhere.
